# Mind the middleman: How receptor-like cytoplasmic kinases mediate plant immunity

**DOI:** 10.1093/plphys/kiad060

**Published:** 2023-02-02

**Authors:** Guadalupe Fernandez-Milmanda, Bernarda Calla

**Affiliations:** Department of Plant Biotechnology and Bioinformatics, Ghent University, 9052 Ghent, Belgium; VIB, Center for Plant Systems Biology, 9052 Ghent, Belgium; United States Department of Agriculture, Agricultural Research Service, Corvallis, OR 97330, USA

Plant perception of non–self-molecules in the extracellular space leads to a general defensive response known as pathogen triggered immunity (PTI) (Jones and Dangl 2006). In the first steps of PTI, pattern recognition receptors (PRRs), which are localized to the cell membrane and contain transmembrane and extracellular domains, bind conserved microbe-derived elicitors known as pathogen-associated molecular patterns or microbe-associated molecular patterns (MAMPs). The signal induced by the receptor-ligand interaction is then amplified by phosphorylation cascades mediated by mitogen-activated kinases (MAPKs) and ultimately leads to the activation of plant defenses. Defense outputs include production of reactive oxygen species and defensive secondary metabolites, stomatal closure, and callose deposition.

Between PRRs and MAPKs is a third class of important molecules: the receptor-like cytoplasmic kinases (RLCKs). RLCKs are considered “sensors or modifiers of sensors” (Liang and Zhou 2018), being neither direct receptors of MAMPs (because they lack extra-cytoplasmatic regions or transmembrane domains) nor necessarily participants of phosphorylation cascades. RLCKs play such a key role in plant immunity that pathogens have evolved mechanisms that directly target them to overcome plant defense and plants have counter-evolved pathogens by expanding their sets of RLCKs (Shiu et al. 2004; Dezhsetan 2017). In the tomato (*Solanum lycopersicum*) genome, there are approximately 128 RLCKs (Sakamoto et al. 2012).

In this issue of *Plant Physiology*, Sobol et al. (2023) characterized a tomato RLCK that interacts with tomato flagellin sensing 2 (Fls2) and flagellin sensing 3 (Fls3), the PRRs that trigger activation of PTI upon detection of flagellin. Therefore, this RLCK was named Fls2/Fls3-interacting receptor-like cytoplasmic kinase (Fir1). The interaction of Fir1 and Fls2/3 was corroborated in a set of experiments including yeast-two-hybrid assays, *in planta* split luciferase assays, and in vitro pull-down assays. Additional in vitro kinase assays with Fir1 and the cytoplasmic domains of Fls2 and Fls3 showed that Fir1 is not a phosphorylation substrate of Fls2 or Fls3, demonstrating that Fir1 is not part of a phosphorylation cascade. Thus, although Fir1 interacts with Fls2/3, the outcome of such interaction requires further research.

Transient expression of Fir1 fused to YFP in *Nicotiana benthamiana* indicated that Fir1 localizes to the plasma membrane, despite the absence of a transmembrane domain. Mutating the potential myristoylation and palmitoylation modification sites in the N-terminus of Fir1 led to the mis-localization of Fir1 protein, suggesting that Fir1 is anchored to the plasma membrane by fatty acid modifications.

To assess the functional relevance of Fir1 in immunity, the authors engineered two CRISPR/Cas9 Fir1 mutants and tested their susceptibility to different microorganisms. Both *fir1.1* and *fir1.2* mutant lines displayed increased susceptibility to the pathogenic bacteria *Pseudomonas syringae* pv *tomato* (*Pst*) DC3000 compared to wild-type (WT) plants. In addition, pretreatment with flagellin-derived MAMPs, which prime defense responses against *Pst* DC3000 in WT plants, did not prime or enhance defenses in *fir1.1* and *fir1.2* mutants. Together, these results suggest that *fir1* mutants are compromised in flagellin-induced immunity.

Next, the authors tested if Fir1 plays a role before or after bacteria penetrate the leaf surface (pre or postinvasion immunity). Bacteria were delivered by either dipping the plants in a bacterial solution, which coats the plant surface with bacteria but requires stomatal penetration, or vacuum infiltration, which forces the entrance of the bacteria into the apoplastic spaces within the leaf and does not require entrance through the stomata. No differences in susceptibility were found after vacuum infiltration. However, when inoculating by dipping, *fir1* lines were more susceptible to *Pst* DC3000 than WT. Furthermore, *fir1* lines failed to close their stomata after bacterial dipping. Thus, Fir1 is required for preinvasion immunity.

Finally, the authors performed a transcriptomic analysis of vacuum-infiltrated plants to evaluate if Fir1 could shape defense signaling. They found that several markers associated with jasmonic acid (JA) signaling were deregulated. JA is a defense hormone that regulates resistance against necrotrophic pathogens and is usually antagonistic of defenses triggered by biotrophic pathogens (Glazebrook 2005; Pieterse et al. 2012) such as those induced by *Pst* DC3000. In agreement with the gene expression data, *fir1* lines were more resistant to the necrotrophic fungus *Botrytis cinerea* compared with their WT counterparts. Therefore, Fir1 could balance the generally antagonistic responses to necrotrophs/biotrophs by regulating JA signaling.

The long-standing battle between plants and pathogens has shaped plant evolution, leading to the generation of numerous tools to perceive, transduce and respond to threats. From this wide arsenal, Sobol et al. have characterized Fir1, a RLCK involved in preinvasion immunity. Although more research is needed to uncover the exact function of Fir1, the results presented in this work reveal that Fir1 participates in flagellin-triggered immunity, interacts with Fsl2 and Fls3, and regulates stomatal opening. Deeper knowledge on the molecular mechanisms governing plant-defense responses allows for a better understanding of evolutionary forces and informs and potentiates research geared toward more efficient and sustainable agriculture.
